# Editorial: Bat-and-ball sports: culture and society

**DOI:** 10.3389/fspor.2024.1381076

**Published:** 2024-02-14

**Authors:** Rogelio Puente-Díaz, Borja García-García

**Affiliations:** ^1^ School of Business and Economics, Anahuac University of North Mexico, Huixquilucan de Degollado, Mexico; ^2^ School of Sport, Exercise, and Health Sciences, Loughborough University, Loughborough, United Kingdom

**Keywords:** bat, ball, cricket–sport, baseball, culture, history

**Editorial on the Research Topic** Bat-and-ball sports: culture and society

Bat and ball sports have a long tradition (our focus is mainly on Baseball and Cricket; See Helyar (1) and Das (2) for a brief history of both sports). The development of these sports has shown and been part of the evolution of relevant social issues such as racism, sexism, and the use of technology and artificial intelligence, to mention a few. Within the evaluation of bat and ball sports, ongoing contradictions and clashes emerge between two forces: tradition vs. modernism. These clashes represent a great opportunity to examine bat and ball sports, their evolution, and the difficulties and challenges experienced by sports managers, commentators, academicians, and fans.

Sports administrators, scholars, and fans often face these clashes and have to make up their minds about whether to support tradition or modernism as presented by different dilemmas. For example, interesting debates have emerged in Cricket to make games shorter and to change the format of tournaments to speed up the pace of games. Players, fans, and administrators who favor tradition claim that the game should remain with similar formats, whereas those favoring modernism argue that audiences want more excitement and a faster pace of games. While we do not intend to discuss the merit of each position, we do seek to use it as an example of the constant clashes between tradition and modernism observed in one of the most popular bat and ball sports.

Another example comes from the suggestion to eliminate umpires in Baseball to call balls and strikes and instead use machines to do the calling. As in the previous example, fans, players, and, administrators favoring tradition support the idea of “leaving the game” as it is, even arguing that errors in strike and ball calls are part of the game. In contrast, those supporting modernism want to see a faster, more precise game with the aid of technology to make more accurate strike-ball calls.

The clash between tradition and modernism represented a great opportunity to make a broad call to scholars to submit their work on different aspects of bat and ball sports. This call was received with enthusiasm and four excellent, insightful articles were accepted for this special issue. Testing the clash between tradition and technology, the article by Christi et al. tested the validity of the lay belief that “sunglass tint” improves catching performance” among Cricket fielders by conducting a laboratory experiment. Results showed that sunglass tint did not significantly influence catching performance. Lay beliefs are held by individuals without proof of truth, making these beliefs pervasive even in the presence of contradictory evidence. This study contributes by emphasizing the importance of empirically testing fielders' lay beliefs about the connection between sunglass tint and catching performance, among other potential contributions.

As suggested by Kwak et al., sports can be a vehicle for social protest and discussion. Race, discrimination, and racism continue to be an important subject of discussion in America. Kwak et al. capitalize on this opportunity to examine how teams and players of four professional leagues: National Basketball Association (NBA), National Football League (NFL), National Hockey League (NHL), and Major League Baseball (MLB), reacted to the death of George Floyd caused by police brutality. Results showed that the reactions of MLB teams were similar to the reactions coming from NBA and NHL teams but differed from the reactions coming from NFL teams. Specifically, whereas NFL teams published longer statements on the death of George Floyd, MLB teams used more negative, stronger words expressing their views on racism and social injustice. MLB teams and players condemned the event and framed it as an example of racism and police brutality, which could be seen as an effort to improve public perception as a response to weak reactions observed and criticized in the past.

Cairney et al. confronted the clash between tradition and modernism with an excellent article on the well-known golden ratio applied to baseball. In this interesting article, the authors tested the implication of the golden ratio of wins and losses as a predictor of championships from 1901 to 2019 and whether the golden ratio was more likely to be observed in eras where there was a balance between offense (batting) and defense (pitching). The calculation of the golden ratio has a long tradition in painting, music, and sculpture and has, to some extent, been explored in sports as well. Empirical results showed a connection between the golden ratio, 61.8% winning percentage, and the championships won but mainly when there was a balance between offense (batting) and defense (pitching) and in some eras only, providing partial support for their hypothesis. Hence, the golden ratio appears to have some implications for understanding and enjoying the game of baseball.

Krishna confronted the clash head-on between tradition and modernism by exploring in a fascinating article how when thinking of the game of Cricket, we usually think of men (erroneously). Yet, women have and continue to play an important role. Adopting a critical perspective, Krishna explains how the role of women has been marginalized, oppressed, and ignored. Cricket, as suggested by the author, is probably one of the most popular sports in India, yet the crucial role of women has been minimized or completely ignored with consequences that are still observed today. Krishna suggests that sports scholars should pay closer attention to the role of women in Cricket in future research.

In sum, this special issue presents four interesting articles covering several research themes. We hope readers enjoy these articles as much as we have enjoyed our role as guest editors. Research on bat and ball sports is thriving and will continue to do so in the future.
